# Supplementary material for: Predictors for participation in mass-treatment and female genital schistosomiasis re-investigation, and the effect of praziquantel treatment in South African adolescents
Source: PLoS Negl Trop Dis. 2024 Mar 27;18(3):e0011798. doi: 10.1371/journal.pntd.0011798 (PMC11020363; doi:10.1371/journal.pntd.0011798)
Supplement: Attachment — Submitted filename: Letter-Comments-from-Reviewers.docx [file pntd.0011798.s001.docx]

Luc E. Coffeng, MD PhD
Guest Editor
PLOS Neglected Tropical Diseases

Thank you for reviewing this manuscript; it is highly appreciated. Kindly see tables below with the responses to the review comments and a description of the changes made.

**Comments from Reviewer 2**

| **General comments from Reviewer 2** | **Answers** |
| --- | --- |
| 1. Line 145: use participants instead of study subject | Thank you. Study subjects was changed to participants |
| 2. Line 138-150- remove the bullets - put them in sentences | Thank you. The bullets were removed and put into a sentence |

**METHODS**

**Reviewer 1**

| **Clarification on Data Collection and Analysis:** | **Answers** |
| --- | --- |
| Please provide clarification on whether the data collection, conducted between 2011 and 2013, was retrospective. Additionally, specify when the analysis was conducted, especially considering the intent to publish in 2023. Discuss the implications of this temporal aspect in the limitation chapter. | Thank you for raising this. This was a primary data that was collected by other scientists through participants’ interviews and gynaecological investigations. The findings of interviews and gynaecological investigations were recorded.  So part of the data was analysed previously and there is still data that needs to be analysed. The statistical analysis of the collected data continues (by me, and the other previous PhD candidates have done the same on other topics). Therefore, this paper if statistically analysing the data that was primarily collected during the period 2011 and 2013 through the participant’s interviews, mass treatment and gynaecological investigations. |
| **FGS Lesion Detection and Data Processing:** |  |
| It remains unclear from the methodological description how FGS lesions were detected, including details on data collection and processing. Specify the instruments used, the individuals performing the examination, and their level of training. Discuss whether images were reviewed by independent specialists and, if not, provide the rationale. Address the implications of these aspects in the results in the limitation chapter. | Thank you for bringing this up. It is now addressed under questionnaires and clinical examination (Line 189 – 192 in tracked document)  The FGS lesions were detected by trained medical doctors, using colposcopy. Quality control was done by image analysis afterwards by independent FGS specialist as the supervisor who has worked in five countries since 1994. |
| **Clarification on Necessary Sample Size (Table 1, Row 3)** |  |
| Elaborate on what the necessary sample size referred to in Table 1, Row 3 represents. Provide details on how this figure was determined and its significance to the study. | Thank you. The details of how these figures were obtained are highlighted under material and method (sample size calculation), (Lines 212 – 223 under material and method). |
| **Reference for Line 190 and Rationale for Sample Size (Line 190):** |  |
| Include a reference for line 190, where the need to study 318 FGS patients and 954 without FGS is mentioned. Provide a more explicit explanation of the rationale behind these specific numbers and how they were derived from the probabilities mentioned in line 190. | Thank you for bringing this important issues. We have revised this information due to unavailability of prior data (Lines 212 – 223 under material and method). |
| **Clarification on Venn Diagram Reference (Line 203):** |  |
| Clarify whether the Venn diagram referenced in line 203 is indeed referring to Figure 2 on page 12. Clearly state this connection in the text to avoid any confusion.  These adjustments will contribute to a more comprehensive and transparent presentation of the study methodology, addressing specific concerns and improving the overall quality of the research. | Thank you. This is addressed, and thank you for bringing this up. |

**RESULTS**

**Reviewer 1**

| **Clarification on "Signs of Urinary Schistosomiasis at Baseline" (Line 211):** |  |
| --- | --- |
| In the methodology section, please provide clarification on what is meant by "signs of urinary schistosomiasis at baseline" (line 211). Specify whether this data was collected during the questionnaire phase or the clinical examination phase. | Thank you for bringing this up. This is revised and is now clear. The word “signs” is removed. The data was collected during clinical examination phase. |
| **Add Year to Line 227:** |  |
| In line 227, please add the year for reference. | Thank you, there year is there (it is 2020). |
| **Table 2 and Table 3 - Confidence Interval and P-Value (Grade 8 - Univariate Analysis):** |  |
| In Table 2 and Table 3, include Confidence Intervals and P-values for Row 2 (Grade 8) in the univariate analysis. Additionally, address missing data in the multivariable analysis columns. Consider reporting this in a separate table or marking the missing data/analysis (e.g., N/A) in the rows. | Thank you. These are the indicator variables and therefore we did not have to include confidence interval and p-Value.  For missing data, as a rule, variables where only included in the multivariable analysis if the p-Value from the crude association was less than 0.2%. |
| **Replace "To Account for a Causal Relationship" with "Correlation" (Line 16, Page 14):** |  |
| On line 16, page 14, replace "to account for a causal relationship" with "correlation" for clarity. | Thank you. This is addressed, and thank you for simplifying this. |
| **Table 3 - Consistency in Data Presentation:** |  |
| In Table 3, ensure consistency in how data is presented. Use a uniform format such as "n=208; 9%" throughout, avoiding variations like "(54/90; 60%)" for the sake of clarity and coherence | Thank you. This is addressed. |

**CONCLUSION**

**Reviewer 1**

| **Rewrite Sentence for Clarity (Line 40, Page 18):** |  |
| --- | --- |
| "It is important to note that different investigators define the investigation findings differently, and that the investigators of gynecological examinations were different in baseline and follow-up studies."  Clarification: Does this refer to the data collection of visual inspections for the presentation of lesions? It is not clear in the methodology how this comes about, and this discussion sentence contributes to the confusion. | Thank you for bringing this up. It is now addressed under questionnaires and clinical examination (Line 189 – 192, Line 42 - 43 in tracked document under discussion) |
| **Clarify Phrasing (Line 47, Page 18):** |  |
| Confusing phrasing: "This supports findings in Zimbabwe." Clarity: "A similar study in Zimbabwe, which investigated [add specific details], reported a result consistent with ours. Where a standard (…)" | Thank you, this has been revised as recommended. |
| **Rewrite Complex Sentence (Lines 78-81, Page 19):** |  |
| "Considering the interplay of various factors influencing participation and the contextual dynamics, the observed outcomes may be subject to nuanced interpretations."  Simplification: Rewrite the sentence for better understanding. | Thank you, the sentence is revised to make it clear. |
| **Specify MDA Target Audience (Discussion Chapter):** |  |
| Add to the discussion chapter details about who the Mass Drug Administration (MDA) should target. Considering the emerging consensus that it should not be limited to school-age children, there is a call to include women and girls in reproductive age, as well as efforts to reach the population that did not attend school. | Thank you. This is addressed and priority given to school-age children and women and girls of reproductive health |
| **Address Methodological Limitations (Methodological Review Reference):** |  |
| Acknowledge and address the methodological limitations referenced in the methodological review. Provide insights into how these limitations may have impacted the study and suggest potential avenues for future research to mitigate these constraints. | Thank you, this is now addressed (Line 181 – 186 under discussion in tracked document) |

**ABSTRACT**

**Reviewer 1**

| **Methods (Lines 37-38):** |  |
| --- | --- |
| 1)Specify the total sample size (n) for sample recruitment.  2) Clarify the statement "a few in September" by providing the actual number and explaining the reasons for the decrease in numbers during September  3) Year of the Study (Line 40): Specify the year of the study to provide a clear timeframe for readers. | Thank you, this is addressed. Only 48 were treated in September. The study was conducted between 2011 and 2013. |
| **Results Section:** |  |
| 4) Organize the results under distinct themes for better clarity:  Prevalence of Schistosomiasis Participation Rates Loss to Follow-up among FGS Lesions Follow-up Findings Factors Influencing Treatment and Follow-up Participation (Mass Treatment & Follow-up Gynaecological Investigation) Challenges in Sample Size for Follow-up Analysis Multivariable Analysis results | Thank you, this is revised. |
| **Clarify Loss to Follow-up (Line 48):** |  |
| Clarify if the 70% loss refers to the total sample size of 2293 or specifically to the 1045 learners with FGS lesions.  Provide the total sample size for the follow-up group and clarify the proportion of grade 12 students in the loss. | Thank you. This is now clear in Lines 48, 25 - 26 and 50 - 51 and Line 56). |
| **Consistent Data Presentation:** |  |
| Present data consistently, using the same format throughout (e.g., "12 (of 19)" and "3 out of 5"). Remove the term "huge" from line 54 for a more objective description. | Thank you. This is revised as recommended. |
| **Improved Conclusion:** |  |
| Revise the conclusion for clarity and impact:  "Despite challenges in sample size and significant loss to follow-up among those with baseline FGS, limiting the ability to fully understand the treatment's effect, multivariable analysis demonstrated a significant treatment effect on abnormal blood vessels." | Thank you. This is revised as recommended. |

**AUTHOR SUMMARY**

**Reviewer 1**

| **Line 76:** |  |
| --- | --- |
| Replace "diagnosed as suffering from diseases such as" with "misdiagnosed with." | Thank you, this has been revised as recommended. |
| **Line 87:** |  |
| Remove the term "huge" for more measured language. | Thank you, this has been revised as recommended. |
|  |  |

**INTRODUCTION**

**Reviewer 1**

| **Reference to WHO Policy Brief (Last Paragraph):** |  |
| --- | --- |
| Add a brief sentence referring to the recently published WHO policy brief titled "Deworming adolescent girls and women of reproductive age. Policy brief." | Thank you. This is added. |
